# Skeletal-dental features in 33 bull terrier dogs

**DOI:** 10.1186/s12917-022-03164-0

**Published:** 2022-02-07

**Authors:** Monica C. Martins, Sara A. Valadares, Jerzy P. Gawor, Lisa A. Mestrinho

**Affiliations:** 1 Faculdade de Medicina Veterinária, Universidade de Lisboa, Lisbon, Portugal; 2 Hospital Veterinário Bom Jesus, Braga, Portugal; 3 Klinika Weterynaryjna Arka, Krakow, Poland; 4 CIISA - Centro de Investigação Interdisciplinar em Sanidade Animal, Faculdade de Medicina Veterinária, Universidade de Lisboa, Lisbon, Portugal

**Keywords:** Dental anomalies_1_, Malocclusions_2_, Klinorhynchy _3_, Bull terrier_4_, Dog_5_

## Abstract

**Background:**

The Bull terrier breed has been reported in the veterinary literature to suffer frequent dental and skeletal malocclusions. In this retrospective case series, we report skeletal-dental anomalies in a group of 33 Bull terriers presented for a dental consultation.

**Results:**

Out of 33 dogs examined, 24 cases had full mouth radiography or Cone-beam computed tomography performed. Eruption and development abnormalities observed were as follows: hypodontia in 54.1% (13/24), eruption changes in 29.2% (7/24), and tooth shape abnormalities in 33.3% (8/24). All dogs presented with some type of dental or skeletal malocclusion: neutroclusion was the most common (66.7% of the animals), followed by mandibular mesioclusion (18.8%), maxillo-mandibular asymmetry (9.4%), and mandibular distoclusion (6.3%). Dental abnormalities noted included rotation of mandibular and maxillary premolar teeth, distal displacement of the incisor teeth, lingual displacement of the mandibular canine teeth, and absence of mandibular premolar and molar teeth. Lingual displacement of mandibular canine teeth was associated with malocclusion causing trauma (odds ratio 7.1, 95% confidence interval [1.4 to 36.1], *p* = 0.024).

**Conclusions:**

Malocclusions and tooth shape abnormalities were found to be the most frequent finding in this group of Bull terriers. Although these findings cannot be generalized to the global population further studies are needed to observe the true expression of these anomalies in the general breed population.

**Supplementary Information:**

The online version contains supplementary material available at 10.1186/s12917-022-03164-0.

## Background

The Bull terrier is an English canine breed, derived from a genetic mix of four breeds, English white terrier, Bulldog, later the Dalmatian, and more recently the Staffordshire bull terrier (American Kennel Club). The Bull terrier breed shows a distinct head shape with a curved profile. The downward pointing of the snout is the principal feature of this breed that distinguishes it from most breeds [1]. This feature is known as klinorhynchy which means the property of a downwardly bent facial skeleton in relation to the cranial base. The resulting angulation between the skull base and the hard palate can lead to skeletal-dental problems. These problems can be with a more exaggerated profile as it can impact the shortening of the jaws. Tooth and occlusion regularities are concerns for most professional breeders. However, the information included in this breed’s standard mentions the need for a complete scissor bite and does not consider numeric changes or other tooth anomalies (American Kennel Club and Federation Cynologique Internationale).

Skeletal malocclusions can result from jaw length discrepancy and dental malocclusions from changes in tooth position, often combined [2]. They can cause potentially serious consequences depending on the type of malocclusion and teeth involved, from mild soft tissue trauma to severe dental and bone lesions. According to previous reports, the Bull terrier breed tends to have frequent dental and skeletal malocclusions [3, 4].

Understanding the most frequent dental anomalies according to a specific breed can help veterinarians achieve early diagnosis and recognize a possible predisposition to acquired dental disease, namely malocclusion causing trauma.

The goal of the study was to report dental anomalies observed in a group of Bull terrier dogs that presented for a dental consultation in two veterinary dental practices.

## Results

Thirty-three Bull terrier dogs were included in this study; 15 were male, 18 were female, 29 were intact, and the four neutered were all females. The average age was 1.1 years (range: 0.5–2 years), and the average weight was 18.6 kg (range: 14.9–22.8 kg). Photographic documentation was obtained in all cases, stone dental models prepared in 23, full-mouth radiography performed in 21, and CBCT performed in five dogs. None of the animals had immediate genetic relation, since no immediate common ancestor were identified; however, older ancestors were not registered.

Eruption and development anomalies were evaluated in 24 cases where there were full-mouth radiographs or cone beam tomography (CBCT) was performed. Hypodontia was observed in 54.1% (13/24). Dental absence was confirmed radiographically in 16 cases (16/24), all occurred in the mandible - in 50.0% (8/16) of the dogs the 4th premolar tooth was absent and in 87.5% (14/16) the 3rd molar tooth was absent. In 62.5% (5/8), the mandibular 4th premolar tooth absence was bilateral, and in 50.0% (8/16), the mandibular 3rd molar tooth absence was bilateral. In 25.0% (4/16) of the dogs, both 4th premolar teeth and 3rd molar teeth were absent bilaterally. Impacted teeth were found on 20.8% (5/24) of the patients. This finding was always observed on the mandibular first premolar tooth.

In 33.3% (8/24), there were tooth shape anomalies. The most frequent anomalies were fused roots in mandibular molar teeth (second and third) and peg shape teeth in fourth mandibular premolar teeth. (Additional File 1).

**Fig. 1:**
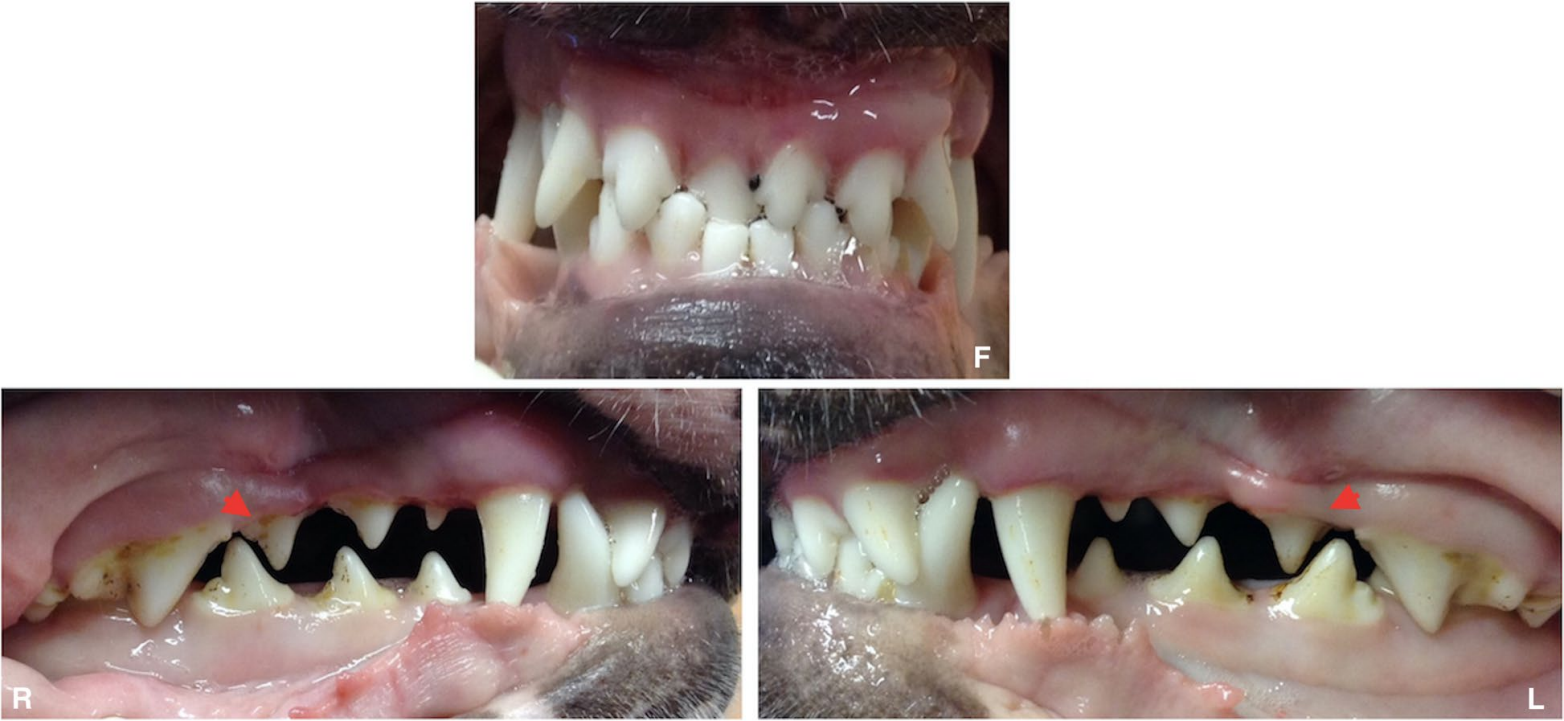
Right (R), frontal (F) and left (L) view of a 2-year-old female Bull terrier dog where it is possible to identify distal displacement of the maxillary third incisors, right maxillary first incisor and canine teeth. Both mandibular canine teeth are occluding lingually and slightly mesially from the normal interdental space. There is also loss of normal interdigitation of maxillary and mandibular premolars and rotation of the maxillary 107 and 207 (red arrows)

**Fig. 2:**
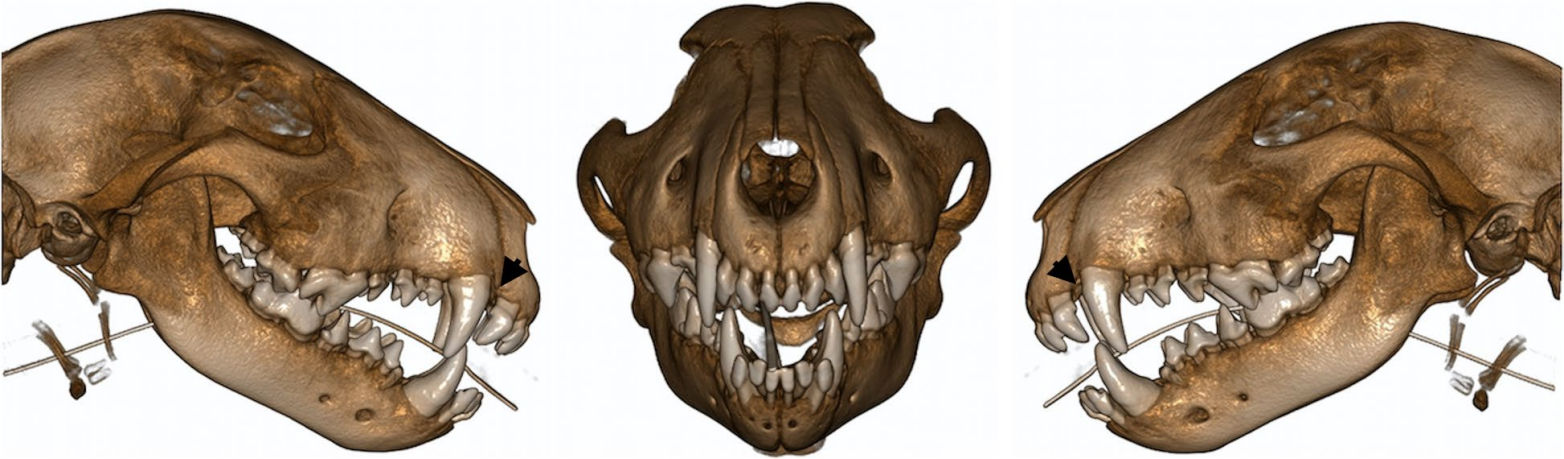
Cone-beam computed tomography three-dimensional reconstruction illustrating the occlusion of a Bull terrier. It is possible to appreciate the narrowing of the maxillary interdental space between third incisor-canine teeth (black arrows) due to distal displacement of the third incisor teeth and lingual displacement of the mandibular canine teeth

Occlusion and tooth position were evaluated in all 33 cases. All dogs presented with some type of malocclusion (Figs. 1 and 2). Class 1 malocclusion (neutroclusion) was the most frequent malocclusion and was found in 66.7% of cases (22/33); Class 2 (mandibular distoclusion) was found in 6.3% of the dogs (2/33). Class 3 (mandibular mesioclusion) was found in 18.8% (6/33), and Class 4 (maxillo-mandibular asymmetry) was found in 9.4% (3/33) of the dogs.

Regarding occlusion anomalies, tooth rotation was the most prevalent, 90.9%, followed by distal, lingual, labial, and mesial displacement of teeth, in 66.7, 60.6, 42.4, and 6.1% of all cases, respectively. Crowding was also frequently found (Figs. 1 and 2).

Sex was not significantly associated with any anomaly (Additional Files 2 and 3). Numeric changes (i.e., decreased number of teeth) were not significantly associated with malocclusion type, shape, crowding, or evidence of malocclusion causing trauma. Malocclusion type was not significantly associated with malocclusion causing trauma, crowding, non-eruption or shape. The presence of a malocclusion causing trauma was not significantly associated with crowding, non-eruption, or shape change (Supplementary File 2). Malocclusion causing trauma was observed in 66.7% (22/33) of the cases and was significantly associated with linguoverted mandibular canine teeth (odds ratio 7.1, 95% confidence interval [1.4 to 36.1], *p* = 0.024).

**Fig. 3:**
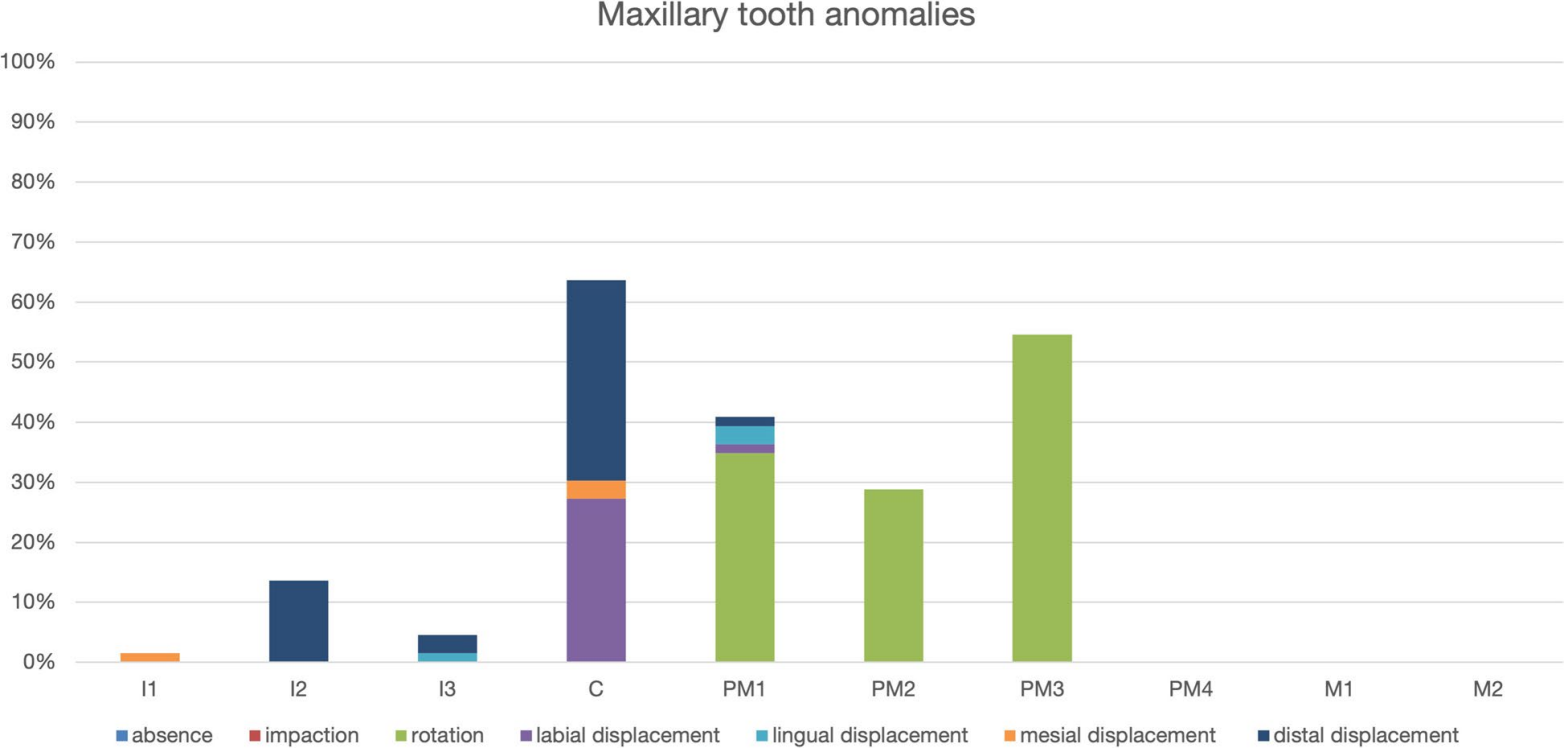
Maxillary tooth anomalies identified in 33 Bull terrier dogs

Regarding maxillary teeth, there was no tooth absence or impaction. Displacement was the most frequent anomaly, namely rotation in premolar teeth (third, second, and first) as well as distal and labial displacement of canine teeth (Fig. 3).

**Fig. 4:**
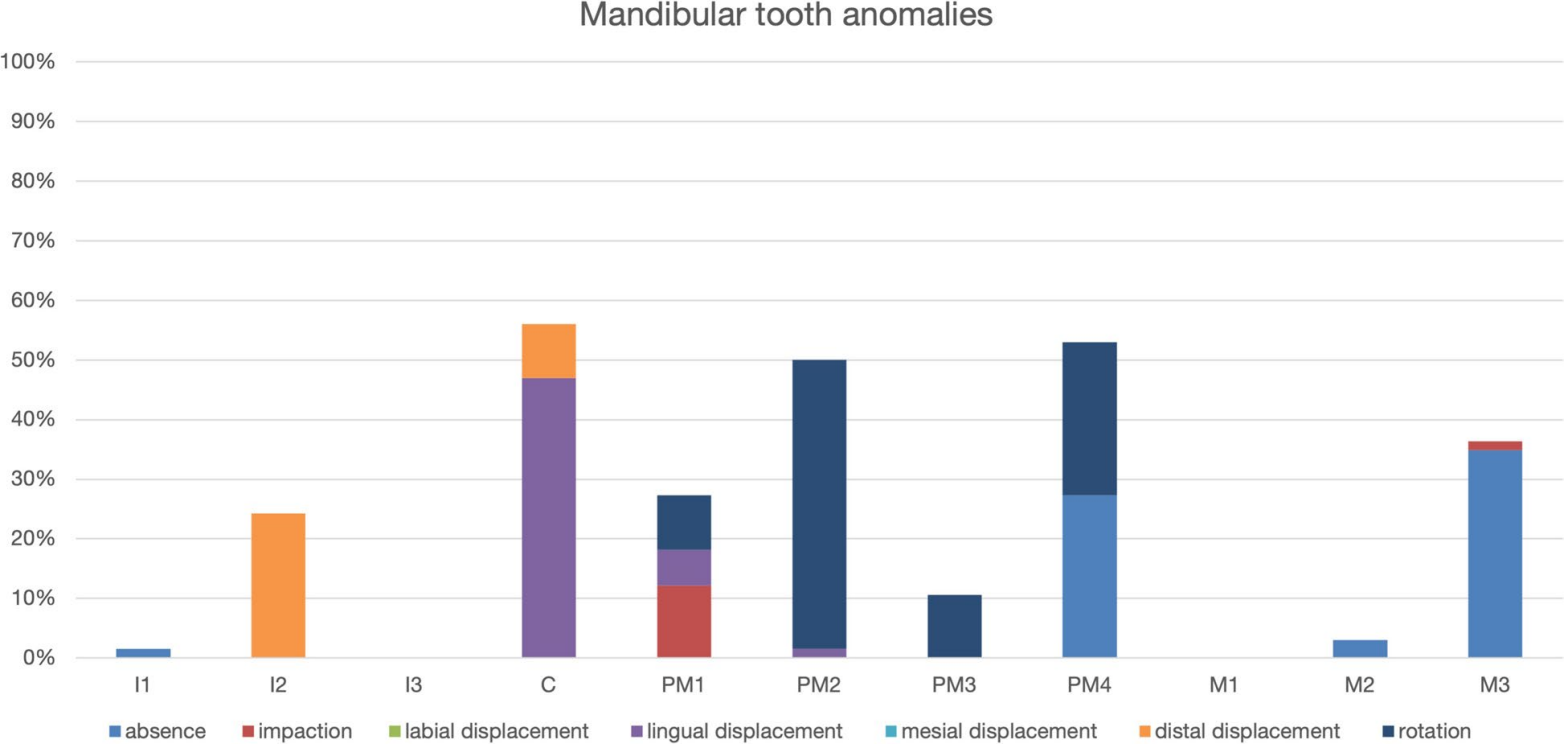
Mandibular tooth anomalies identified in 33 Bull terrier dogs

Regarding mandibular teeth, tooth absence was primarily observed in the mandibular third molar teeth and the mandibular fourth premolar teeth (Fig. 4). Impaction was primarily observed in mandibular first premolar teeth. Lingual displacement was primarily observed in mandibular canine teeth and distal displacement in mandibular second incisor teeth. Rotation was primarily observed in the second premolar tooth (Fig. 4).

## Discussion

Observational studies of skeletal-dental changes in specific breeds are rare and to the authors’ knowledge are nonexistent in the Bull terrier breed. Although, anecdotal skeletal-dental anomalies in the Bull terrier have been reported by other authors [3, 5].

In the group of dogs studied, dental absence confirmed radiographically was found in half of the cases, always in mandibular teeth. Due to the young age of these animals and the absence of a history of trauma and dental treatment, these were likely cases of dental agenesis, or complete absence. Tooth shape anomalies were observed in a quarter of cases, most namely peg shape teeth of the 4th mandibular premolar teeth and fused roots of the second mandibular molar teeth. This type of shape anomaly corresponded to the most frequent location of agenesis in this group of dogs, again, 4th mandibular premolar teeth and 3rd mandibular molar teeth. This observation suggests a tendency for fewer teeth adjusted to the reduction in mandible length. However, malocclusions and dental anomalies do not follow Mendelian heritage, meaning that other factors can influence the final occlusion outcome [6, 7].

Regarding eruption changes, the mandibular 1st premolar tooth was the most frequently impacted/embedded tooth. This anomaly is frequently found in brachycephalic dogs [8–10], with one study reporting this finding in the Bull terrier [10]. Again, crowding and the maxillo-mandibular discrepancy can contribute to this event [11].

In the present study, all dogs presented some type of malocclusion. In almost two-thirds of the cases, the malocclusion caused trauma to the oral cavity. Although skeletal neutroclusion was observed in two-thirds of cases, from tooth rotation to distal to lingual tooth displacement. Crowding was also found in most cases (*n* = 25). Mesioclusion was the most frequent skeletal malocclusion (18.8%), followed by maxillo-mandibular asymmetry and distoclusion.

It was not possible to identify significant associations between most of the variables studied, however we observed a significant number of cases of malocclusions causing trauma, significantly associated with lingual displacement of mandibular canine teeth. These observations reinforce that shortening of the maxilla with subsequent maxillo-mandibular disarrangement limits the access of the mandibular canine teeth to the expected interdental maxillary space. Persistent deciduous teeth are the most frequently reported explanation for this type of malocclusion, causing narrowed mandibular canine teeth positioning [12]. However, in this case series, such an anomaly was not observed. It is theorized that the lingual displacement of mandibular canine teeth results from either one or a combination of causes, including a primary narrow mandible or a reduced maxillary interdental space between the maxillary third incisor tooth and maxillary canine tooth. Acknowledgment of these anomalies can increase the awareness from clinicians to identify problems at an early stage and correct them. Furthermore, these findings should be considered in the selection of breeding specimens because this anomaly might occur more frequently in more extreme klinorhynchy skulls.

The Bull terrier breed occurs frequently for orthodontic evaluation (author’s observations), suggesting that there might be a predisposition. The low heterozygosity of this breed was observed in a genetic diversity study that concluded that Bull terriers, among other breeds, should be improved in terms of genetic variability [13]. The low genetic diversity associated with its unique skull features may carry an intrinsic risk of presenting with a malocclusion, especially in more extreme klinorhynchy skull conformations where maxilla-mandibular balance could be disturbed. The klinorhynchy conformation causes curving of the incisive bone which leads to distoversion of the maxillary incisors, particularly the third incisor. Consequently, the incisors can pose an obstacle to the mandibular alignment causing the entrapment of the mandibular canine teeth lingually behind the third incisors. The current study observed consistent dental occlusion deviations that could result from this conformation: a shortened maxilla length, reduced interdental space between the third maxillary incisor tooth and the canine tooth and a narrow mandible.

Limitations of this study include the study design and number of animals. The design was observational and based on a referral caseload from two dentistry facilities. Therefore, an intrinsic bias precludes generalization of the results to the general population of Bull terrier dogs and limits the calculation of the true incidence of these anomalies. Due to the absence of individuals with common lineage, it was impossible to establish any heredity patterns. The overall number of evaluated individuals was small; the former may have led to underestimating tooth shape anomalies and possibly limiting the observation of any other associations. A more significant number of individuals and a more standardized procedure (full-mouth radiographs or CBCT) would have strengthened the results of this study.

## Conclusion

In conclusion, dental and occlusion anomalies were frequently found in this group of Bull terriers. The most prevalent observations were rotation of mandibular and maxillary premolar teeth, distal displacement of the maxillary incisor teeth, lingual displacement of the mandibular canine teeth, and absence of mandibular 3rd molar and 4th premolar teeth. These findings cannot be generalized to the worldwide population of Bull terriers and further studies are needed in order to evaluate the true occurrence of these anomalies in this breed.

## Methods

This was a retrospective study. We analyzed clinical records of Bull terriers presenting to two veterinary practices [location blinded for review] between January 2017 and January 2021.

Inclusion criteria included Bull terrier pedigree, less than 2 years old, no history of trauma, no other diseases or prior dental treatment, complete history, dental chart performed under anesthesia, and availability of at least one of the following: documentation of 3-view oral photography, dental stone models, dental radiography, or cone-beam computed tomography (CBCT). Immediate ancestor, but not older ancestors, was registered.

We recorded clinical variables (age, sex, weight) and dental-skeletal anomalies according to the American Veterinary Dental College nomenclature. Dental anomalies (number, impaction, shape, and position) and skeletal anomalies (class 1 - neutroclusion, class 2 - mandibular distoclusion, class 3 – mandibular mesioclusion, and class 4 - maxillo-mandibular asymmetry). Dental malocclusions were assessed by evaluating each tooth individually (mesio-, disto-, palatal-, linguo-version, and rotation) [14].

Results were registered using commercial software (Microsoft Excel for Mac version 16.49) used for the descriptive statistical analysis. A second commercial software package was used for inferential statistics (IBM SPSS Statistics for Mac version 26). The chi-square or Fisher’s exact test was performed to evaluate the association between categorical variables (sex, presence/absence of numeric changes, malocclusion type, presence/absence of shape changes, crowding, or malocclusion causing trauma). A *p*-value < 0.05 was considered significant for a 95% confidence interval.

## Supplementary Information

**Additional file 1.** Sample characterization.

**Additional file 2.** Analysis of the associations between sex and different variables studied.

**Additional file 3.** Analysis of the associations between eruption changes and other variables.

## Data Availability

The original contributions presented in the study are included in the article. Further inquiries may be directed to the corresponding author.
