# Supplementary material for: Skeletal-dental features in 33 bull terrier dogs
Source: BMC Vet Res. 2022 Feb 7;18:65. doi: 10.1186/s12917-022-03164-0 (PMC8819867; doi:10.1186/s12917-022-03164-0)
Supplement: Supplementary file 1 — Additional file 1. Sample characterization. [file 12917_2022_3164_MOESM1_ESM.docx]

Table 1 – Sample characterization

|  |  | *n* |  |
| --- | --- | --- | --- |
| Age |  |  | 1.14±0.5 years |
| Weight |  | 33 | 18.6±6.97 Kg |
| Sex | **F** | 33 | 18 |
|  | **M** | 33 | 15 |
| Full mouth X-ray |  | 24 | 21 |
| CBCT |  |  | 5 |
| Malocclusion type | **1** | 33 | 22 |
|  | **2** |  | 2 |
|  | **3** |  | 6 |
|  | **4** |  | 3 |
| Malocclusion causing trauma | **present** | 33 | 22 |
|  | **absent** |  | 11 |
| Crowding | **present** | 33 | 25 |
|  | **absent** |  | 8 |
| Rotation | **present** | 33 | 29 |
|  | **absent** |  | 4 |
| Numeric changes | **decreased** | 24 | 17 |
|  | **no change** |  | 7 |
|  | **increased** |  | 0 |
| eruption changes | **impacted** | 24 | 7 |
|  | **no** |  | 24 |
| shape changes | **present** | 24 | 8 |
|  | **absent** |  | 17 |
