# Supplementary material for: Skeletal-dental features in 33 bull terrier dogs
Source: BMC Vet Res. 2022 Feb 7;18:65. doi: 10.1186/s12917-022-03164-0 (PMC8819867; doi:10.1186/s12917-022-03164-0)
Supplement: Supplementary file 2 — Additional file 2. Analysis of the associations between sex and different variables studied. [file 12917_2022_3164_MOESM2_ESM.docx]

Table 1 – Analysis of the associations between sex and different variables studied.

|  |  | Sex | |  |
| --- | --- | --- | --- | --- |
|  |  | **M** | **F** | **p value*** |
| Maloclusion type N=33 | **I** | 10 | 12 | 0.520 |
|  | **II** | 0 | 2 |  |
|  | **III** | 3 | 3 |  |
|  | **IV** | 2 | 1 |  |
| Maloclusion causing trauma N=33 | **present** | 9 | 13 | 0.488 |
|  | **absent** | 6 | 5 |  |
| Crowding  N=33 | **present** | 11 | 14 | 1 |
|  | **absent** | 4 | 4 |  |
| Rotation  N=33 | **present** | 14 | 15 | 0.607 |
|  | **absent** | 1 | 3 |  |
| Numeric changes N=24 | **decrease** | 6 | 7 | 0.078 |
|  | **no change** | 1 | 10 |  |
|  | **increase** | 0 | 0 |  |
| Eruption changes N=24 | **impacted** | 5 | 2 | 0.182 |
|  | **no** | 2 | 11 |  |
| Shape changes N=24 | **present** | 5 | 3 | 0.390 |
|  | **absent** | 6 | 10 |  |

*Fisher exact test or Pearson chi-square when applicable

Table 2 – Analysis of the associations between eruption changes and other variables.

|  |  | Eruption changes | |  |
| --- | --- | --- | --- | --- |
|  |  | **Decreased** | **No change** | **P value*** |
| Angle maloclusion N=33 | **I** | 9 | 9 | 0.176 |
|  | **II** | 2 | 2 |  |
|  | **III** | 4 | 4 |  |
|  | **IV** | 2 | 2 |  |
| Traumatic maloclusion N=33 | **present** | 12 | 4 | 0.647 |
|  | **absent** | 5 | 3 |  |
| Crowding  N=33 | **present** | 12 | 4 | 0.647 |
|  | **absent** | 5 | 3 |  |
| Shape anomalies N=24 | **present** | 6 | 2 | 1 |
|  | **absent** | 11 | 5 |  |
| eruption changes N=24 | **present** | 7 | 7 | 0.065 |
|  | **absent** | 10 | 0 |  |

*Fisher exact test or Pearson chi-square when applicable

Table 3 – Analysis of the associations between malocclusion type and other variables.

|  |  | Malocclusion type | | | |  |
| --- | --- | --- | --- | --- | --- | --- |
|  |  | **I** | **II** | **III** | **IV** | **p value** |
| Traumatic maloclusion N=33 | present | 15 | 0 | 4 | 0 | 0.137 |
|  | absent | 7 | 0 | 4 | 0 |  |
| Crowding  N=33 | present | 16 | 2 | 4 | 3 | 0.577 |
|  | absent | 6 | 0 | 2 | 0 |  |
| Shape anomalies N=24 | present | 6 | 1 | 1 | 0 | 0.682 |
|  | absent | 10 | 1 | 3 | 2 |  |
| eruption changes N=24 | present | 2 | 1 | 2 | 2 | 0.041 |
|  | absent | 14 | 1 | 2 | 0 |  |

*Fisher exact test or Pearson chi-square when applicable

Table 4 – Analysis of the associations between malocclusion causing trauma and other variables.

|  |  | Malocclusion causing trauma | | |
| --- | --- | --- | --- | --- |
|  |  | **present** | **absent** | **p value*** |
| Crowding  N=33 | present | 17 | 8 | 1 |
|  | absent | 5 | 3 |  |
| Shape anomalies N=24 | present | 4 | 4 | 0.363 |
|  | absent | 12 | 4 |  |
| Eruption changes  N=24 | present | 6 | 1 | 0.352 |
|  | absent | 10 | 7 |  |

*Fisher exact test or Pearson chi-square when applicable
